# Evaluation of a Paper-Based Checklist versus an Electronic Handover Tool Based on the Situation Background Assessment Recommendation (SBAR) Concept in Patients after Surgery for Congenital Heart Disease

**DOI:** 10.3390/jcm10245724

**Published:** 2021-12-07

**Authors:** Carolin Rehm, Richard Zoller, Alina Schenk, Nicole Müller, Nadine Strassberger-Nerschbach, Sven Zenker, Ehrenfried Schindler

**Affiliations:** 1 Department of Anesthesiology, Catholic Children’s Hospital Wilhemstift, 22149 Hamburg, Germany; 2 Staff Unit for Medical and Scientific Technology Development & Coordination, Coordination (MWTek), Commercial Directorate, University Hospital Bonn, University of Bonn, 53127 Bonn, Germany; Richard.Zoller@ukbonn.de (R.Z.); zenker@uni-bonn.de (S.Z.); 3 Department of Medical Biometry, Informatics and Epidemiology, University Hospital Bonn, University of Bonn, 53127 Bonn, Germany; Alina.Schenk@ukbonn.de; 4 Department for Pediatric Cardiology, University Hospital Bonn, University of Bonn, 53127 Bonn, Germany; Nicole.Mueller@ukbonn.de; 5 Department of Anesthesiology and Intensive Care Medicine, University Hospital Bonn, University of Bonn, 53127 Bonn, Germany; N.Nerschbach@hotmail.com

**Keywords:** electronic checklist, paper-based checklist, handover OR to PICU/ICU, SBAR, PDMS

## Abstract

(1) Background: we compare a new SBAR based electronic handover tool versus a paper-based checklist for handover in a pediatric intensive care unit (PICU). (2) Methods: this is a randomized, observational study of 40 electronic vs. 40 paper checklist handovers after pediatric cardiac surgery, with a 48 items checklist for comparison of reporting frequencies and notification of disturbances and noise. PICU staff satisfaction was evaluated by a 12-item questionnaire. (3) Results: in 14 out of 40 cases, there were problems with data processing (incomplete or no data processing). Some item groups (e.g., hemodynamics) were consistently reported at higher frequencies than other groups. Items not specifically asked for did not get reported. Some items, automatically processed in the SBAR handover page, did not get reported. Many handovers suffered a noisy and distracting atmosphere. There was no difference in staff satisfaction between the two handover approaches. Nurses were highly unsatisfied with the general approach by which the handover was performed. (4) Conclusions: human error appears to be a main factor for unreliable data processing. Software is still too complicated, and multitasking is a stressful and error prone event. Handover is a complex task with many factors required for a successful completion.

## 1. Introduction

Patient handovers, defined as: “the transfer of information and professional responsibility and accountability between individuals and teams,” are high-risk, error-prone patient care episodes [1,2]. The transfer of patient information can be affected by poor communication and teamwork, unstable patients, interruptions, distractions, technical problems with pumps, ventilators or monitoring, inconsistent teams, and poor standardization [1,2,3]. Handover without a protocol leads to omission of important information and inconsistent information [4]. We do know that standardized checklists improve handover accuracy [5,6,7]. Handovers, especially in pediatric cardiac intensive care units, have been investigated and show, after implementation of a standardized handover protocol, a reduction in errors, decrease in technical problems and improvement of team work and communication, hence increasing patient safety [6,8,9,10]. There are signs of fewer post-operative adverse events related to enhanced communication and information transfer [9]. The implementation of a standardized handover protocol seems to be sustainable, with good handover results even after the post-intervention phase [11], and a team hand-off approach leads to less omission of information, improves efficiency, and increases staff satisfaction [12].

In the last decade, the Situation Background Assessment Recommendation (SBAR) communication tool from the submarine duty hand off by the US Navy, got introduced into medical handovers and has been reported to improve communication between nurses and doctors [13,14]. There is some evidence suggesting that SBAR increases patient safety, but robust clinical study evidence is lacking, especially on patient outcomes and adverse events [15].

With digitization and more electronic data processing in hospitals, there is a growing need for electronic handover checklists, which realize the potential of electronic documentation systems for structured reuse of patient data to improve clinical processes. Currently it remains unclear whether electronic handover tools are superior to paper-based checklists. Therefore, there is need for investigation and comparison of these methods [1,16].

There are different types of checklists. The type most commonly used in anesthesia, so called “shopping list” checklists, primarily serve as memory aids. Because of real-time monitoring and the implementation of more electronic devices, e-checklists have been reported to be useful [16]. They are supposed to enhance communication and data transfer, compared to a paper- based checklist. Recall of information from memory is often inaccurate and leads to mistakes and loss of information, favoring electronic data transfer [3,5]. Doctors and nurses believe that electronic devices and patient records will improve patient safety, quality, and effectiveness of work, as well as communication processes [17,18].

While electronic handover tools introduce additional complexity and, thus, inevitable new failure modes and challenges in human technology interaction. The advantages of data accuracy, real-time data transfer, completeness, and timeliness, favor this future prospective [1].

To our best knowledge, this is the first prospective randomized study comparing the use and implementation of a standardized electronic handover tool presenting structured, patient specific data to electronically support a standardized handover, based on the SBAR concept, with a conventional paper-based checklist. The aim of this study is to exploratively compare the two different handover methods, to identify problems in either handover protocol and thus pave the way towards more user acceptance for new interventions and future devices that provide electronic handover support that is more accurate, safer, and more convenient to use.

## 2. Materials and Methods

### 2.1. Ethic Statement

This study has been approved by the ethics board of the University Hospital of Bonn, Germany as a randomized, observational quality control study, with anonymous data collection and no need for written consent of patients or participating staff.

### 2.2. Background

Handover from operating room (OR) to PICU used to be performed with a paper-based checklist, which has been used for about ten years quite successfully. Since the hospital is on its way towards completely paperless processes, which, for anesthesia and intensive care units (ICU), are implemented primarily using an electronic patient data management system (PDMS) (ICM 10.01 by Drägerwerk AG & Co. KGaA, Lübeck, Germany) wherever possible, there was need for a new handover concept.

For ICU documentation, as well as the anesthesia protocol in the OR, the PDMS automatically transfers, hemodynamic measures, as well as ventilator settings, into the electronic reports. Other data, such as medication, fluids, lines, tubes, and others have to be chosen out of a menu and confirmed manually to get transferred into the record.

### 2.3. Electronic Handover Tool Implementation and Paper Checklist

For this study, we adapted a preexisting SBAR based handover protocol, designed primarily for use in adult patients and being rolled out across adult perioperative medicine at the study site at the time of inception of this study. To make it applicable for pediatric cardiac surgery patients, many items were added and adjusted to make it useful for caregivers and comparable to the previously used paper-based checklist. This process included members of the PICU, anesthetics, and the clinical IT team responsible for PDMS configuration development and maintenance the electronic handover tool is structured along the four major areas of the SBAR concept: situation, background, assessment, and recommendation. There are a total of 26 input fields or checkboxes. Content for 11 of these fields is automatically pre-completed from the electronic anesthesiologic documentation and can be accepted as part of the definitive documentation, adjusted, or deleted with a click of the mouse. In another three fields, entries should be completed by the responsible anesthesiologist in the operating room (preoperative anamnesis and diagnostics, instructions from the anesthetist, and additional information).

This document is called up for handover at the patient’s bedside in the PICU. All entries from the OR are now available. The transfer follows the SBAR structure and thus, the structure of the page. Every item that appears in the list is discussed and clicked on. It starts with the patient’s identity. If this is mentioned, the corresponding checkbox is ticked off. If contents from the OR that can already be read as text from the OR are mentioned in the text fields, these are also clicked on and thus confirmed and, if necessary, supplemented. Dedicated input fields for each professional group involved (anesthetist, surgeon, nurse) are provided (e.g., operation history). In this way, a complete handover report is created, which is also available at any other point in time.

The pre-existing paper-based checklist to which the handover tool was compared, consists of a DIN A4 paper form being a typical “shopping list” checklist, containing fields for name, diagnosis, operation performed, surgeon, lines, catheters, drains, medication, catecholamines, hemodynamics, blood loss, blood substitution, clotting substitutes, labs, temperature, and CBP-Times. On the backside of the paper, surgeons had the possibility to draw sketches, to visualize the performed operation. It was kept bedside and was accessible to everybody at any time.

### 2.4. Study Design

We compared the two handover protocols by having an investigator observe OR to PICU handovers, who documented coverage of crucial handover items using a study checklist. The checklist contained patient history and demographic data (9), lines and tubes (7), intra-operative history (18), medications (6), drains and wounds (4), and disturbances (4). Items were ticked positive if they were verbally communicated. Furthermore, we looked for disturbances such as bleeps, phone calls, patient instability, or unrest and side talks. Side talks were noted as being disturbing when the observer had problems following the handover due to noise.

The current workflow for handovers in PICU is described as follows: surgeon, anesthetist, and the anesthetic nurse bring the patient from the OR to his or her place in the PICU. Handover is performed from anesthetist to intensivist and consultant intensivist, who are following the checklist, while the responsible nurse is checking lines, drawing blood, and connecting the ventilator, monitor, and pumps. He or she gets a handover from the intensivist after accommodating the patient.

We investigated the perceived handover quality and satisfaction of doctors and nurses with the handover process by a simple 12 item questionnaire and an optional comment section to add their own opinions or suggestions. There were three descriptive questions on job, age, and work experience. One item asked for the kind of handover performed. Eight items concerning satisfaction with handover, were rated on a 10-point Likert scale, from 0 “do not agree” to 10 “fully agree”.

### 2.5. Implementation and Data Collection

The participating colleagues (4 surgeons, 11 anesthetist, 11 intensivists, about 30 PICU nurses) were trained in using the PDMS and were introduced to the use of the SBAR protocol within the PDMS. The paper-based checklist was already well known to everyone and did not need any extra training. After two weeks dry run using the PDMS SBAR handover in August 2020, the data collection started, taking place from September 2020 until January 2021. We observed 80 handovers in total, randomized to Group 1 “PDMS” (*n* = 40) and Group 2 “paper-based checklist” (*n* = 40). Randomization was done by “random.org”. The handovers were observed by a certified clinical trial assistant and an anesthetist, who was part of the study team and worked in the pediatric cardiac anesthesia team. They were called depending on availability. Due to the nature of intensive care unit staffing and shift work, some handovers and questionnaires might have been performed and answered more than one time by the same person, which was not taken into account in the analysis.

About thirty minutes before the end of surgery, the anesthetist in charge called one of the two observers to get to know which study protocol was applying. This information was passed on to PICU, while informing of the patient’s expected arrival. In case of complete failure of the PDMS, participants were encouraged to use the paper-based checklist. When the handover was finished, the questionnaire was handed out to the intensivist and the nurse responsible for the patient. They were asked to answer the questions as soon as possible and to return the questionnaire immediately.

### 2.6. Statistics

Statistics were performed by using SPSS software (IBM Version 27 Corp., Armonk, NY, USA). Frequencies mean and percentages were calculated for descriptive comparison. We used Fisher’s two-tailed exact test, two-tailed t test for mean equality, Wilcoxon–Mann–Whitney-U-Test for independent samples and paired Wilcoxon-Test for dependent samples, where applicable. A *p*-value of <0.05 was considered statistically significant. Analysis is of exploratory; hence *p*-values were unadjusted.

## 3. Results

### 3.1. Handover Comparison

A total of 80 handovers were observed. In Group 1, 14 out of 40 cases experienced problems with the PDMS, while in Group 2, no problems were observable, due to the fact that no PDMS was used (Table 1). In one case, there was a hardware problem with the anesthesia machine in the OR, and it had to be replaced with a machine not being integrated in the PDMS. We are assuming that, in 13 cases, the anesthetist did not fully complete and confirm the anesthesia record, as well as the SBAR page, by ticking the confirmation button (a procedurally required quality assurance step designed to prevent transmission of invalid data). If these tasks are not completed, the data will not, or just incompletely be transferred. In five cases, there was no data transfer whatsoever, so the paper-based checklist applied. These cases have been excluded from further analysis, as well as two handovers in Group 1 (PDMS) where both checklist methods were used simultaneously.

Further calculations were performed with a total of *n* = 73 after excluding the seven aforementioned cases: 33 handovers remained in the PDMS group and 40 in the paper-based checklist (Table A1). Eight patients were in PICU prior to surgery and handover, evenly spread with four in each group.

In only 74% of the handovers, the patient’s identity was verbally verified. The handover of the patient’s age and weight was remarkably low with 50 and 42%, considering the study population being from new born to adolescent, where accurate weight and age are very important for calculating and administering drugs or ventilator settings. The handover of the diagnosis for surgery was observed in 76.7%, whereas the performed surgery was communicated in 90.4%. The only two items standing out with larger differences in relative frequencies between the groups were allergies (39.2% vs. 12.5%) and medication prior to operation (57.6% vs. 10%), being reported more often in Group 1 (PDMS). These two items were specifically asked for in the PDMS SBAR page but not in the paper- based checklist.

Insertion site and type of IV lines and tubes were overall reported the most frequently (79.5–93.2%). Notable differences were only reached for Foley catheter (66.7% vs. 27.5%) with more reports in Group 1 (PDMS), whereas “tube size and depth” was reported more frequently (78.8% vs. 95%) in the paper-based checklist. Foley catheter was included in the PDMS SBAR handover page, whereas the tube size was not automatically transferred by the PDMS, but it was specifically asked for in the paper checklist.

Within the “intraoperative surgery” section, the items—hemodynamic situation (87.7%), transfusion (97.3%), blood clotting and substitution (86.3%), POCT and labs (89.6%), diuresis (64.4%), peculiarities (68.5%), and time for questions (87.7%)—had high reporting frequencies and were consistent within both groups. Ventilator settings (66.7% vs. 27.5%) and anesthesia recommendations (33.3% vs. 7.5%) were handed over more frequently in the PDMS group, whereas the cardio-pulmonary-bypass (CPB) related items, such as CPB times (57.6% vs. 85%), modified ultrafiltration (18.2% vs. 72.5%), and minimal temperature (33.3% vs. 65%) were communicated more frequently in the paper-based checklist. Considering that not every patient gets a CPB dependent surgery or a modified ultrafiltration, it is important, nonetheless, to report that information. CPB times and minimal temperature were explicitly asked for in the paper checklist, but they were automatically transferred in the PDMS SBAR protocol. There were consistently low reports of fluid balances and applications of crystalloids, maybe because CPB fluid balancing is difficult to estimate, since the anesthetist has no insight in the CPB record in this setting. The contribution of extubated patients (50.7%) was even in both groups. Ventilator settings and anesthesia recommendations had a box to tick in the PDMS SBAR page to complete the handover protocol.

Handover of current medication was very frequent (80.8–94.5%) for all but antiemetics (27.4%), which are more likely to be administered in fast track patients. Sedatives were reported less frequently in the PDMS group (75.8% vs. 95%), probably because they are not asked for in the PDMS handover, but neither are analgesics.

Surgery details were mentioned more often in the paper-based checklist, especially for the “possibility to ask question”. Surgeons were more often present at the paper handovers (Table A1), which may explain this finding.

In 53.4% of the cases, social disturbances and side talks during the handover made the handover difficult to follow. Mobile phones or bleeps went off in 15.1%, and patient instability or unrest in 21.9% of the handovers, causing interruptions.

In summary, most of the items showing different reporting frequencies between the groups were specifically asked for in one checklist but not in the other. For example, in the PDMS checklist, there were specific fields asking for allergies, previous medications, ventilator settings, and recommendations, whereas in the paper checklist, there were fields asking for CPB times, temperature, modified ultrafiltration, and fast track medications. Surgery details were consistently reported more often in the paper-based checklist. Surgeons attended paper-based handovers significantly more often, being at hand for questions (Table 2). Overall, the differences in the reporting frequencies were to be expected, given that the respective checklist asked for specific items.

It is quite interesting, however, that some items automatically transferred in the PDMS SBAR protocol are mentioned more often than others, and some have low reporting frequencies. The Foley catheter gets more handover reporting in the PDMS than in the paper checklist, even though it is specifically asked for in the paper checklist. On the other hand, CPB times, being asked for in both handovers, are omitted more often in the PDMS group, even though the information is displayed on the monitor.

Average time for handover was calculated in 70 cases, with three missing because of incomplete data (Table A2). Mean duration was 10.2 min, ranging from 4 to 25 min. The PDMS handover, on average, took approximately one minute longer than the paper-based handover. The difference was not statistically significant.

Part taking staff members were calculated in 73 handovers, with an average of 7 people present at the handover (range 4–10). There was no difference between the two groups (Table A3).

Some of the investigated items such as “patient instability or unrest” or “social disturbances” might be confounded by whether the patient was extubated or not. Therefore, patient extubation/intubation rates were examined (Table A4). The distribution of extubated and intubated patients was similar between the groups. A total of 37 patients were extubated (50.7%), with 19 (57.6%) in the PDMS group and 18 (45%) in the paper-based group (*p* = 0.35) (Table A1).

Allergies were more often reported in extubated patients, as well as information related to the intubation. The hemodynamic situation was more reported in the group with intubated patients. This might be the cause for not being fast tracked, or they simply represent high-risk surgical patients, such as neonates.

There were different reporting rates for analgesics and antiemetics between the groups, frequently seen in the extubated fast track patients. Social disturbances and side talks occurred at similar rates in both groups, while patient instability or unrest was observed slightly more often in the fast track patients, but it was not statistically significant between the groups. There appeared to be no relation to a noisy surrounding or patient instability, and the fact that a patient was fast tracked or not.

Average duration of handovers (*n* = 70) in extubated patients was 9.8 min. compared to 10.6 min. in intubated patients (Table A5). This difference was likely due to the effect of additional information being reported, such as the ventilator settings, installing more pumps, more unstable hemodynamic situations, and a higher-risk operation. Nevertheless, the difference was not statistically significant.

### 3.2. Questionnaire

**Figure 1:**
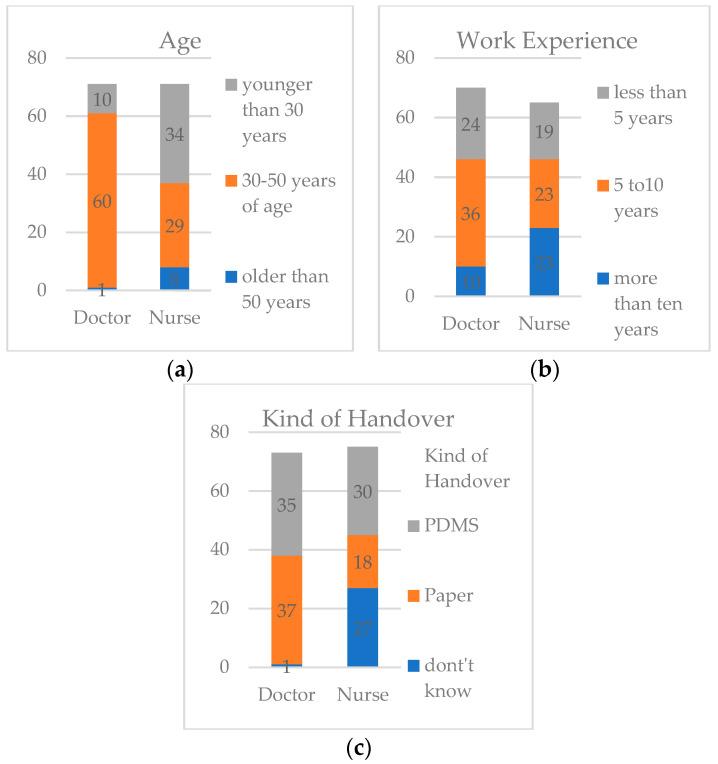
(**a**) Staff age distribution in PICU; (**b**) Staff work experience in years in PICU; (**c**) Types of handover performed in PICU.

There were 93.8% (*n* = 75) of the nurses and 91.3% (*n* = 73) of the intensivists who returned their questionnaire (Figure 1). Nurses’ age distribution was 47.9% for under 30 years, 40.8% for 30–50 years, and 11.3% above 50 years of age. For the doctors, the majority was between 30 and 50 years (84.5%), with 14% being under 30 years, and only 1.4% (*n* = 1) being above 50 years of age.

Work experience of the nurses was less than five years in 29.2%, 5–10 years in 35.4%, and more than 10 years in 35.4%. About half of the intensivists (51.4%) had work experience of 5–10 years, 34.3% had less than 5 years, and 14.3% had more than 10 years. 

The distribution of different age groups and work experience within the two groups were similar and not statistically significant. Interestingly, 36% of the nurses did not know which handover was performed.

**Figure 2:**
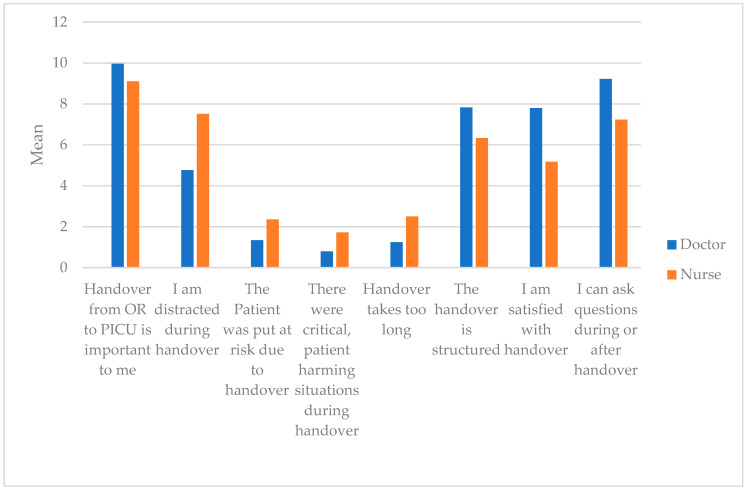
Questionnaire on quality and satisfaction with handover performed. Comparison of Doctors and Nurses. (OR = operation room, PDMS = patient data management system, PICU = pediatric intensive care unit).

Generally speaking, the handover is very important to all participants in the PICU (Table A6). Distraction during handover is an issue, but it mainly affects nurses. The impact of distraction is much higher on them, due to multitasking and settling the patient. Nonetheless, there was no belief that patients were put at risk during handover because of distraction. Neither was it a belief that patient harming or critical situations occurred. The duration of handover was considered to be adequate, whereas the structure of the handover process could be improved, and nurses, especially, favor a different approach. This is reflecting the question of “Satisfaction with handover”. Nurses, on average, tend to be less satisfied, while doctors seem to be quite content with the handover. The possibility to ask questions is given more often for doctors being involved in the communication process, while nurses are busy with the patient and do not get the possibility to engage actively in the handover. Therefore, they lack the possibility to ask questions. Between nurses and doctors, discrepancies for almost all questions are observable. Note that, due to the explorative setting of this project, *p*-values are not adjusted. (Table A7, and Figure 2).

**Figure 3:**
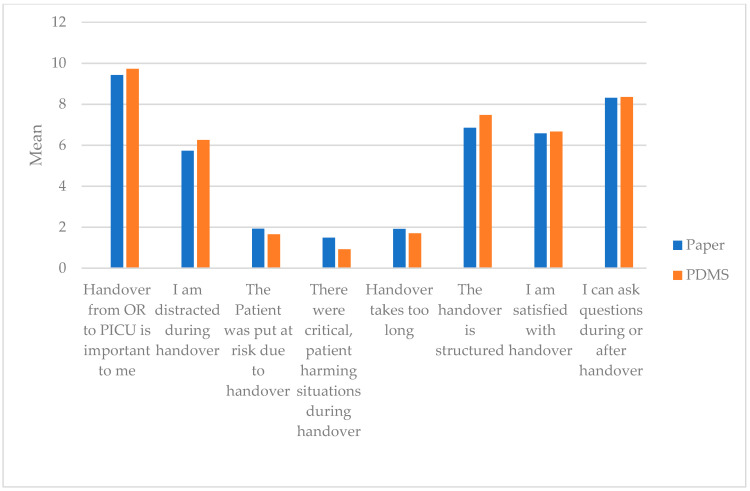
Questionnaire on quality and satisfaction with handover performed. Comparison of electronic PDMS approach and paper-based checklist. (OR = operation room, PDMS = patient data management system, PICU = pediatric intensive care unit).

The two different handover methods seem to have no influence on distraction, satisfaction with handover, risk-prone events, duration and structure of handover, or the subjective possibility to ask questions. Only “Handover from OR to PICU is important to me” showed a difference between the two groups. This was an interesting finding, as the question was of one’s opinion, not relating to the handover approach (Table A8, and Figure 3).

The questionnaire included a comment section where participants were encouraged to suggest their own ideas for improving the handover process or to provide their feedback on the current approach.

There were 15 doctors and 24 nurses who commented in this section. Mainly, doctors asked for better structure and more space, as well as more data transfer (e.g., patient history, medications) in the electronic handover report. Overall, nurses wanted to be more engaged in the handover process. They favored a “team time out” step, where everybody listened, and handover was carried out in a quiet and focused atmosphere.

## 4. Discussion

Electronic handover has its advantages in real-time data transfer [1], hence mitigating the effects of inconsistent reports from memory recall that are prone to error [3,5]. On the other hand, there is always the possibility of hardware failure and technical, or user associated, problems when creating data [1]. We showed that the process of transferring data from OR to PICU was not reliable when using the current implementation of the handover tool, and user mistakes seem to be the major cause of these problems, suggesting a need for significant improvements with regard to both the technical implementation and the usability of the current solution. The task of confirming data in the PDMS to process it on to PICU takes a few focused steps. In our setting, this has to be performed at the time of a pediatric cardiac patient’s transfer from operating table to their bed, together with pumps for medications, such as catecholamines and sedatives, as well as the monitoring device. In our setting, half of the cases are extubated, and the remaining cases need hand assisted ventilation. This is a very critical moment in patient’s care and represents a moment full of tasks and stresses for the anesthetist who is required to multitask. One has to question whether this is the best time to complete computer confirmation, unless steps for data confirmation are made much simpler. Stressful working environments should be avoided to increase patient safety [19]. In addition to usability improvements, a revision of the procedural setup, for example, enabling the anesthetist to prepare most of the handover documentation prior to patient transfer, could contribute to mitigating this challenge.

Developing electronic devices or software is usually done by personnel who do not use it in real time or are familiar with the situation, therefore close collaboration with clinical staff is necessary. There is a need for improving checklist design and usability of specific fields they are used in [20,21]. In our setting, the staff was involved in the development and checklist design of the SBAR page, but they had to make compromises concerning space and clarity, since it was supposed to be used on all ICU’s, including (adult ICU’s) of the university hospital, as well. As is typical of clinical IT projects, an agile approach to development and evolution, where insight from practical use informs the next iteration of development, is expected to be beneficial, for which the results of this study provide important input.

Additionally, we found that, even though important information, such as CBP times, was displayed in the electronic handover protocol, participants saw no need in reporting this obvious information, possibly because they are of no interest to their own profession and are, therefore, not relevant to them. We also cannot exclude that participants felt that the display and documentation of these data in the electronic handover protocol was sufficient for communication, obviating the need for explicit verbal communication. On the other hand, we found demographic data, such as “Patient name” or “Diagnosis”, had poor reporting frequencies in both handover groups, with an average of 75%, although they are specifically asked for. The groups were comparable and seemed to suffer the same problem: poor team and communication skills.

SBAR improves communication strategies and team performances, as well as team communication skills between doctors and nurses, which increase patient safety. However, it does require team training for its proper usage and communication [13,14]. There is some evidence that SBAR does not improve memory and recall of information [22], and the compliance to use the tool is not always high, due to items anesthetists consider not to be relevant. To increase user compliance, it could be helpful to add the option of “not applicable” in the checklists [23].

Checklists, regardless of the type, acquire data they are specifically asking for. Our findings indicate that, if checklists miss out crucial items, those tend to be reported less frequently, just as if no checklist is used. Hence, one should create specific checklists adapted to the need of the setting they are used for [4]. A rigid SBAR approach might not always be the best way, especially for highly specialized handovers, but it is a good line to follow [14,23].

We know that electronic checklists are better than no checklist, but it remains questionable whether they are superior to paper checklists [1,5]. As we can show, there is not much evidence so far. The information exchange is often thought to improve with electronic checklists compared to paper checklists, especially as displayed items get reported more often [8]. With this current exploratory study, we can only partly support this notion (e.g., CBP times). It seems that electronic checklists, compared to paper checklists, reduce errors and workload for staff, but they do not reduce time for completion. It is not clear whether they increase patient safety [24].

In our findings, we could not show that the clinical staff were in favor of one type of handover over the other. Electronic checklists seem to be a more efficient and focused handover, functioning as cognitive aid of intra-operative information, and the acceptability of these tools by the teams seem to be high. Information processed is found again later if needed, therefore hopefully reducing adverse events and increasing patient safety [18,21,24]. That requires complete, reliable data transfer and access for everybody who might need to use the information. Having insight in the report during handover is important, which requires big screens, tablets, or paper handouts. Digitized data may not be available to all users, limiting its application. Another limitation with electronic checklists is their inflexibility to allow entry of individual notes or drawings, as well as limited space for comments [20]. Our findings support this statement. Surgeons seem to be more satisfied with the conventional paper-based approach. This may be due to more space and, possibly, for describing and drawing surgery details on paper, which is not possible in the current electronic format used in this study.

Handover of patient information is a task that usually involves a group of clinical staff from different teams and specialties. Depending on the experience of the staff and their specialties, the information passed on is more or less focused on subjective importance and knowledge, leading to inconsistency [4]. Usually standardization of handover is poor and there is no teamwork or problem focused communication. Checklists help standardize these procedures, improve handover accuracy, reduce errors, increase patient safety, and enhance communication and teamwork [1,5,6,7,9,25,26]. Measuring the outcome of adverse events of poor handovers is difficult [27]. Many of these checklists have their background in aviation and have been adopted in several medical settings, especially in OR, as with “team time out” procedures and SBAR handover concepts. Anesthesia is the medical specialty with the most use of checklists and evaluation of them. As real-time monitoring and implementation of more electronic devices are anticipated in future medicine, electronic checklists are likely to become more relevant and useful [5].

Our findings suggest that both methods, as currently implemented, are equivalent to each other, with the advantage of real-time data transfer favoring the electronic handover process with regard to future prospective. The possibility of processing more patient data [28], such as patient history and CPB records, makes it especially more comprehensive and easy to use, and user acceptability will, therefore, increase [5]. Intraoperative handover is prone to error and false transmission of information [29]. This can be minimized by an electronic record and handover, improving patient safety and enhancing teamwork and communication [5]. We also found that an electronic handover tool needs to be more customized for specific needs—in this case, to a pediatric cardiac intensive care ward. Doctors perceive the workflow of the SBAR based PDMS handover page as not very convenient in this setting and that it requires some remodeling to better suit their needs and to keep the communication flow going. There is a need for more space to write notes. There may be too much unnecessary information being processed, which needs to be straightened out. Sometimes, data processing from another program in the PDMS SBAR page takes too long (POCT), so it is not processed into the SBAR page, thus leading to missed information on PICU. Some processes, such as data confirmation in the OR, could be less prone to errors.

In summary, the current implementation of the handover tool was largely perceived by users as equivalent to the established paper checklist, with significant room for future improvement, particularly with regards to usability, thus supporting the potential to create an electronic solution clearly superior to the well-established paper approach in user perception, as well as functionality.

### 4.1. Handover Performance

Patient handover in ICU is a “high risk error-prone patient care episode” [2], which is characterized by multitasking with machines, pumps, ventilators and monitors, inconsistent teams, unstable patient condition, complex patient history, and noise [30]. Interruptions and distractions during handover lead to poor team performance [31] and may increase the risk of patient morbidity and mortality [2,3]. Factors for good handovers are clear information, good communication skills, and a quite focused atmosphere [19]. As indicated here, handover, in general, is very important to doctors and nurses, but there is a high incidence of interruptions and noise in the observed handovers, regardless of the handover methods. Probably, the result of staff not being actively engaged in the handover and, therefore, being dissatisfied by the approach the handover is performed. The nurses report that they are very distracted during handover, that they lack the possibility to ask questions—compared to the doctors—and that they are not satisfied with the handover performance. They are not included in the communication process, and they are busy with patient accommodating tasks, which requires talking and communication itself. This makes the whole handover process very noisy and distracting. Most of the nurses are required to be more actively involved. Doctors, on the other hand, seem to be quite content with the structure and the way the handover is performed, no matter which protocol applies. Doctors and nurses judge the duration of the handover as adequate, and they do not believe the handover causes critical or patient harming situations. A new tactic with a “sterile cockpit” and a “Team Time Out” step, which the nurses are favoring, might lead to a quiet, private surrounding, diminishing disturbances and the expected participation of the complete team [1]. Team time out or “hands off” is an essential aspect of effective team communication and delivery of information, improving team performance, minimizing interruptions, and improving quality of handover [10,23]. Hand-off procedures lead to more concentration and less multitasking [32]. This might require team training in practical and communication skills [1,2]. Team training of the handover process, such as in aviation or formula one, reduces errors in handover and improves transfer of information [33]. To improve post-op handover communication, partaking staff should be present at handover with the ability to ask questions [1,2]. One should “encourage a team approach and engagement of all parties during the hand off process diminishing communication barriers placed by role and seniority of different care personnel” [3].

### 4.2. Limitations

In this study, we focused on the verbal communication process of handover in a quantitative way. Some items might not have been reported because they simply did not apply, and we did not double check with the patient records. Therefore, it is possible some items were underreported. Similarly, some data were processed but were not verbally communicated. There was no check on the quality of the item statement. The two checklists were not 100% comparable, hence we found specific sections that had better reporting frequencies than others, outlining that the process of implementing an electronic checklist was promising but was not completed yet.

Depending on observer availability, we mainly had to exclude handovers that were performed after normal working hours. The majority of handovers took place on regular workdays between 12 am and 5 pm. Hence, we probably missed more severe cases with long surgery, one or two intra-operative handovers between anesthetists, and, most likely, patient instability during handover, as well as more tired clinical staff. These cases could be even more vulnerable to communication errors, misinformation, and missing data. Due to the nature of working hours and shifts in OR and PICU, it was not possible to randomize participating staff. The second observer, an anesthetist himself and part of the study team, has been taking active part in some of the handovers himself, because of manpower distribution within the hospital, and thus might be biased. There is a likelihood for a Hawthorne effect, since the anesthetists could feel observed and judged by their handover, hence trying to be as precise as possible. We did not investigate and measure the outcomes of the patients or the impact of the handover on adverse events. For further development of the checklist, there should be more “active users” involved, such as surgeons, which might increase user acceptance. All results are based on explorative and descriptive analysis, and they require further investigation in future studies that may take our results into account.

## 5. Conclusions

Take the best of each: a combination of the two methods would be the golden path. A rigid SBAR approach leaves information out that is crucial for complex patient histories. Therefore, we recommend a more flexible development of checklists, so they can be adjusted for specific needs. Electronic data transfer is the future in medicine, and the implementation of these tools needs to be accompanied by an iterative improvement cycle to gain user acceptance. It has to be easy to use and reliable. Missing data is not acceptable, and human error is an important factor, which can be partially mitigated by usability optimization. Last but not least, poor team performance, interruptions, and noise are key factors for poor handover. Nobody involved in patient care should be left out in a transfer of information. A team time out approach and team training in handover processes might be the key, for which robust and usable technical solutions may provide relevant support.

## Figures and Tables

**Table 1:** Technology and data transfer problems.

	Total *n* = 80		PDMS = 40		Paper = 40		
	Total	%	*n*	%	*n*	%	Fisher’s Exact Test
Technology	80	100.0%	40	100.0%	40	100.0%	
PDMS Problems	14	17.0%	14	35.0%	0	0.0%	<0.000 *
No Data Transfer	5	6.3%	5	12.5%	0	0.0%	0.055

* = *p* value < 0.05 (PDMS = patient data management system).

**Table 2:** Attending staff at PICU handover.

	Total *n* = 73		PDMS *n* = 33		Paper *n* = 40		
Attending Staff	Total	%	*n*	%	*n*	%	Fisher’s Exact Test
PICU Supervising Intensivist	65	87.7%	29	87.9%	36	90.0%	1.000
PICU Intensivist	73	100.0%	33	100.0%	40	100.0%	
PICU Nurse 1	73	100.0%	33	100.0%	40	100.0%	
PICU Nurse 2	67	91.8%	30	90.9%	37	92.5%	1.000
Other	16	21.9%	7	21.2%	9	22.5%	1.000
Surgeon	54	74.0%	20	60.6%	34	85.0%	0.031 *
Anesthetist	73	100.0%	33	100.0%	40	100.0%	
Anesthetic nurse	52	71.2%	22	66.7%	30	57.7%	0.450

* = *p* value < 0.05 (PDMS = patient data management system, PICU = pediatric intensive care unit).

## Appendix A

**Table A1:** Comparison of handover reports: PDMS vs. Paper.

	Total *n* = 73		PDMS *n* = 33		Paper *n* = 40		
	Total	Percent	Quantity	Percent	Quantity	Percent	Fisher’s Exact Test
Demographics	73	100.0%	33	100.0%	40	100.0%	
Name	54	74.0%	26	78.8%	28	70.0%	0.434
Age	36	49.3%	17	51.5%	19	47.5%	0.816
Weight	31	42.5%	13	39.4%	18	45.0%	0.644
Diagnosis	56	76.7%	28	84.8%	28	70.0%	0.170
Surgery	66	90.4%	31	93.9%	35	87.5%	0.446
Allergies	18	24.7%	13	39.4%	5	12.5%	0.013 *
Patient History	46	63.0%	20	60.6%	26	65.0%	0.809
Medication	23	31.5%	19	57.6%	4	10.0%	<0.000 *
Multi-Resistant Germs	2	2.7%	2	6.1%	0	0.0%	0.201
**Tubes and Lines**							
Arterial Line	67	91.8%	29	87.9%	38	95.0%	0.400
Central Venous Line	68	93.2%	29	87.9%	39	97.5%	0.169
Bladder Catheter	33	45.2%	22	66.7%	11	27.5%	0.001 *
Venflons	61	83.6%	26	78.8%	35	87.5%	0.357
Problems during Induction	25	34.2%	12	36.4%	13	32.5%	0.807
Intubation	58	79.5%	27	81.8%	31	77.5%	0.774
Tube (size/depth)	64	87.7%	26	78.8%	38	95.0%	0.069
**Intraoperative History**							
Ventilator settings	33	45.2%	22	66.7%	11	27.5%	0.001 *
Hemodynamic	64	87.7%	29	87.9%	35	87.9%	1.000
FastTrack attempted	21	28.8%	8	24.2%	13	32.5%	0.604
Extubation	37	50.7%	19	57.6%	18	45.0%	0.350
Transfusion	71	97.3%	31	93.9%	40	100.0%	0.201
Blood clotting and Substitution	63	86.3%	27	81.8%	36	90.0%	0.332
POCT, Labs, ACT	72	98.6%	32	97.0%	40	100.0%	0.452
Blood loss	53	72.6%	21	63.6%	32	80.0%	0.187
Fluid Balance	9	12.3%	5	15.2%	4	10.0%	0.723
Crystalloids	19	26.0%	5	15.2%	14	35.0%	0.065
Diuresis	47	64.4%	21	63.6%	26	65.0%	1.000
CPB Times	35	47.9%	6	18.2%	29	72.5%	<0.000 *
Modified Ultrafiltration	37	50.7%	11	33.3%	26	65.0%	0.01 *
Minimal Temperature	53	72.6%	19	57.6%	34	85.0%	0.016 *
Anaesthesia Events	34	46.6%	14	42.4%	20	50.0%	0.638
Recommendations Anaesthesia	14	19.2%	11	33.3%	3	7.5%	0.007 *
Peculiarities	50	68.5%	21	63.3%	29	72.5%	0.456
Time for Questions	64	87.7%	30	90.9%	34	85.0%	0.499
**Medications**							
Antibiotic	69	94.5%	31	93.9%	38	95.0%	1.000
Catecholamines	67	91.8%	30	90.9%	37	92.5%	1.000
Protamine	59	80.8%	27	81.8%	32	80.0%	1.000
Sedatives	63	86.3%	25	75.8%	38	95.0%	0.036 *
Analgesics	62	84.9%	28	84.8%	34	85.0%	1.000
Antiemetics	20	27.4%	9	27.3%	11	27.5%	1.000
**Surgery Details**							
Drainages	56	76.7%	22	66.7%	34	85.0%	0.095
Pacemakers	32	43.8%	11	33.3%	21	52.5%	0.155
TOE	46	63.0%	19	57.6%	27	67.5%	0.467
Time for Questions	50	68.5%	18	54.5%	32	80.0%	0.025*
**Disturbances During Handover**							
Social/Side Talks	39	53.4%	19	57.6%	20	50.0%	0.638
Mobile or Bleep	11	15.1%	8	24.2%	3	7.5%	0.057
Patient Instability or Unrest	16	21.9%	8	24.2%	8	20.0%	0.778
PDMS Problems	9	12.3%	9	27.3%	0	0.0%	<0.000 *

* = *p* value < 0.05; (PDMS = patient data management system, POCT = point of care test, ACT = activated clotting time, CPB= cardio-pulmonary-bypass, TOE = transesophageal echocardiography).

**Table A2:** Time for handover performed and attending staff.

	*n*	Minimum	Maximum	Mean	Std. Deviation
Handover in Minutes	70	4	25	10.19	3.67
Attending Staff	73	4	10	6.85	1.28

**Table A3:** Comparison of time for handover performed and attending staff: PDMS vs. Paper.

		*n*	Mean	Std. Deviation	Mann-Whitney- U
Handover in Minutes	PDMS	30	10.77	4.33	0.324
	Paper	40	9.75	3.08	
Attending Staff	PDMS	33	6.67	1.34	0.220
	Paper	40	7	1.22	

(PDMS = patient data management system).

**Table A4:** Comparison of handover performed for fast-tracked and intubated patients (major differences).

	Total *n* = 73		Extubated *n* = 37		Intubated *n* = 36		
	Total	Percent	Quantity	Percent	Quantity	Percent	Fisher’s Exact Test
**Demographics**	73	100.0%	37	100.0%	36	100.0%	
Allergies	18	24.7%	15	40.5%	3	8.3%	0.002 *
**Tubes and Lines**							
Intubation	58	79.5%	34	91.9%	24	66.7%	0.01 *
**Intraoperative History**							
Ventilator settings	33	45.2%	17	45.9%	16	44.4%	1.000
Hemodynamic	64	87.7%	29	78.4%	35	97.2%	0.028 *
Blood clotting and Substitution	63	86.3%	29	78.4%	34	94.4%	0.085
**Medications**							
Catecholamines	67	91.8%	32	86.5%	35	97.2%	0.199
Analgesics	62	84.9%	35	94.6%	27	75.0%	0.024 *
Antiemetics	20	27.4%	18	48.6%	2	5.6%	<0.000 *
**Disturbances During Handover**							
Social/Side Talks	39	53.4%	20	54.1%	19	52.8%	1.000
Patient Instability or Unrest	16	21.9%	11	29.7%	5	13.9%	0.157

* = *p* value < 0.05.

**Table A5:** Comparison of time for handover performed: fast-tracked vs. intubated patients.

		*n*	Mean	Std. Deviation	Mann-Whitney-U
Handover in Minutes	Extubated	35	9.77	3.47	0.456
	Intubated	35	10.6	3.87	

## Appendix B

**Table A6:** Questionnaire on quality and satisfaction with handover performed.

	*n*	Mean	Std.-Deviation	Minimum	Maximum	Percentile		
						25	50 (Median)	75
Handover from OR to PICU is important to me	148	9.55	1.342	1	10	10.00	10.00	10.00
I am distracted during handover	149	6.28	3.440	0	10	4.00	8.00	9.00
The Patient was put at risk due to handover	148	1.75	2.499	0	10	0.00	1.00	2.75
There were critical, patient harming situations during handover	149	1.20	2.284	0	10	0.00	0.00	2.00
Handover is too long	146	1.82	2.530	0	10	0.00	0.00	3.00
Handover is structured	139	7.08	2.857	0	10	5.00	8.00	9.00
I am satisfied with handover	146	6.18	3.298	0	10	4.00	7.00	9.00
I can ask questions during or after handover	147	8.06	2.936	0	10	8.00	9.00	10.00

(OR = operating room, PICU = pediatric intensive care unit).

**Table A7:** Questionnaire on quality and satisfaction with handover performed. Comparison of Doctors and Nurses.

		*n*	Mean	Std. Deviation	Paired Wilcoxon-Test
Handover from OR to PICU is important to me	Nurse	74	9.14	1.79	0.000 *
	Doctor	74	9.96	0.26	
I am distracted during handover	Nurse	75	7.77	2.35	0.000 *
	Doctor	74	4.76	3.71	
The Patient was put at risk due to handover	Nurse	74	2.16	2.83	0.050 *
	Doctor	74	1.34	2.05	
There were critical, patient harming situations during handover	Nurse	75	1.60	2.43	0.024 *
	Doctor	74	0.80	2.07	
Handover is too long	Nurse	72	2.40	2.58	0.002 *
	Doctor	74	1.24	2.36	
Handover is structured	Nurse	65	6.23	3.10	0.005 *
	Doctor	74	7.82	2.41	
I am satisfied with handover	Nurse	72	4.51	3.37	0.000 *
	Doctor	74	7.80	2.28	
I can ask questions during or after handover	Nurse	73	6.89	3.54	0.000 *
	Doctor	74	9.22	1.47	

* = *p* value < 0.05; (OR = operating room, PICU = pediatric intensive care unit).

**Table A8:** Questionnaire on quality and satisfaction with handover performed. Comparison of PDMS and Paper.

Handover PDMS or Paper		*n*	Mean	Std. Deviation	Mann-Whitney-U
Handover from OR to PICU is important to me	PDMS	74	9.74	0.845	0.04 *
	Paper	74	9.35	1.683	
I am distracted during handover	PDMS	75	6.57	3.205	0.416
	Paper	74	5.97	3.660	
The Patient was put at risk due to handover	PDMS	74	1.57	2.410	0.395
	Paper	74	1.93	2.587	
There were critical, patient harming situations during handover	PDMS	75	0.93	2.062	0.097
	Paper	74	1.47	2.473	
Handover is too long	PDMS	74	1.77	2.636	0.531
	Paper	72	1.86	2.434	
Handover is structured	PDMS	70	7.26	2.842	0.511
	Paper	69	6.90	2.881	
I am satisfied with handover	PDMS	73	6.12	3.249	0.677
	Paper	73	6.23	3.369	
I can ask questions during or after handover	PDMS	73	8.08	3.040	0.538
	Paper	74	8.04	2.850	

* = *p* value < 0.05; (PDMS = patient data management system, OR = operating room; PICU = pediatric intensive care unit).
